# Robust Ultrafast Projection Pipeline for Structural and Angiography Imaging of Fourier-Domain Optical Coherence Tomography

**DOI:** 10.3390/diagnostics14141509

**Published:** 2024-07-12

**Authors:** Tianyu Zhang, Jinpeng Liao, Yilong Zhang, Zhihong Huang, Chunhui Li

**Affiliations:** Centre for Medical Engineering and Technology (CMET), School of Science and Engineering, University of Dundee, Dundee DD1 4HN, UK; txzhang@dundee.ac.uk (T.Z.); j.y.liao@dundee.ac.uk (J.L.); y.z.z.z.h.zhang@dundee.ac.uk (Y.Z.); z.y.huang@dundee.ac.uk (Z.H.)

**Keywords:** optical coherence tomography (OCT), optical coherence tomography based angiography, OCTA, angiography projection, OCTA projection

## Abstract

The current methods to generate projections for structural and angiography imaging of Fourier-Domain optical coherence tomography (FD-OCT) are significantly slow for prediagnosis improvement, prognosis, real-time surgery guidance, treatments, and lesion boundary definition. This study introduced a robust ultrafast projection pipeline (RUPP) and aimed to develop and evaluate the efficacy of RUPP. RUPP processes raw interference signals to generate structural projections without the need for Fourier Transform. Various angiography reconstruction algorithms were utilized for efficient projections. Traditional methods were compared to RUPP using PSNR, SSIM, and processing time as evaluation metrics. The study used 22 datasets (hand skin: 9; labial mucosa: 13) from 8 volunteers, acquired with a swept-source optical coherence tomography system. RUPP significantly outperformed traditional methods in processing time, requiring only 0.040 s for structural projections, which is 27 times faster than traditional summation projections. For angiography projections, the best RUPP variation took 0.15 s, making it 7518 times faster than the windowed eigen decomposition method. However, PSNR decreased by 41–45% and SSIM saw reductions of 25–74%. RUPP demonstrated remarkable speed improvements over traditional methods, indicating its potential for real-time structural and angiography projections in FD-OCT, thereby enhancing clinical prediagnosis, prognosis, surgery guidance, and treatment efficacy.

## 1. Introduction

Optical coherence tomography (OCT) is a noninvasive imaging modality, utilizing the principles of low-coherence interferometry to generate high-resolution, three-dimensional visualizations of biological tissues [1,2,3,4]. The development of Fourier-domain OCT (FD-OCT) has broadened its scope by offering enhanced resolution, sensitivity, and scanning speed compared to time-domain OCT (TD-OCT) [5]. FD-OCT can be further divided into spectral-domain OCT (SD-OCT) and swept-source OCT (SS-OCT) [3,5]. The high sensitivity and stability of FD-OCT are more suitable for high-speed imaging compared to TD-OCT [5]. OCT-based angiography (OCTA) is an extended function of OCT imaging, which can provide detailed microvascular networks of tissue in vivo [6,7,8,9,10]. By utilizing OCT imaging’s high resolution and fast acquisition, OCTA can extract OCT signals’ variations caused by moving red blood cells [7]. Current clinical applications of OCTA predominantly revolve around ophthalmology, where OCTA has improved the diagnosis and monitoring of retinal diseases, including age-related macular degeneration, diabetic retinopathy, and vascular occlusions [6,9,11,12]. Besides ophthalmic applications, specialized probe designs extended its applications to other areas, such as dermatology and dentistry [13,14,15,16,17]. 

The fundamental scanning component of conventional OCT scans is the amplitude scan (A-scan or A-line), which represents the back-scattered light intensity across the depth at one location on the tissue surface [18]. While moving the scanning location along a line on the tissue surface, one brightness scan (B-scan or B-frame) as a 2D array can be formed by successively acquiring a number of A-lines. Hence, a cross-section or tomogram of the tissue can be presented as one B-frame [18,19]. A 3D volume of the OCT dataset can be achieved by moving the scanning location on two axes on a line-by-line basis, which can acquire a number of B-frames, forming a 3D volume [18,19]. OCTA datasets are in a 4D volume format with three spatial dimensions and one temporal dimension, which would only require an additional step of repeated scanning at the same location [6,7,20,21]. 

The projections of both OCT and OCTA are 2D matrixes generated from compressing the 3D volumes on the depth axis, which are often used in biomedical research [18,22,23,24,25,26,27,28,29]. However, generating OCT and OCTA projections from raw interference signals can be extremely time consuming [30,31], as explained below. The current method to generate OCT and OCTA projections for FD-OCT requires Fourier transform (FT) [32] firstly to transform the interference signals to intensity signals [24]. After applying FT to all A-line signals, the 3D volume data can be applied with projection algorithms, such as summation projection, to generate the structural OCT projections [29]. For OCTA projection, firstly the input would be 4D volumes. Then, angiography signals can be extracted from either the repeated cross-sectional frames (B-frames) or the whole 4D dataset by using OCTA reconstruction algorithms such as Speckle variance (SV) [33], A-scans eigen decomposition (aED) [20], B-scans eigen decomposition (bED) [21], and windowed eigen decomposition (wED) [30]. Since the temporal dimension is used for OCTA reconstruction, the data format would be in 3D volumes containing the angiography signals only. Lastly, the 3D OCTA data can generate the 2D OCTA projections by using projection algorithms [29].

The structural OCT projections can provide a comprehensive perspective on tissue microarchitecture with many applications including diagnostics, surgical guidance, data segmentation, and therapeutic monitoring [22,23,24,25,26]. The OCTA projection is an intuitive, commonly used method to present the 3D OCTA dataset although the projection process is a reduction in dimensionality [20,21,29,30,31,34]. The vasculature network can be quantitatively analyzed from the OCTA projections, including the vessel diameter, the vessel density, the vessel tortuosity, and other metrics that can be used for clinical research [27,28]. Therefore, OCTA projections can be used for many clinical applications including disease assessment, diagnosis, and image-guided surgery.

The current projection methods for FD-OCT as described above are often processed as one of the postprocessing steps, due to the long processing time [30,31]. Specifically, FT and the 3D angiography reconstruction are the main reasons for the slow processing. In addition, varied reconstruction algorithms can involve iterations on different levels. The clinical use of OCT or OCTA pursues a high efficiency from the start of the data acquisition to the output of results, which would not align with the slow projection methods. A real-time structural projection technique was developed, which would require modifications in the hardware setup, e.g., the laser source [35]. Wei et al. implemented a GPU-accelerated system to provide real-time OCTA projections [36]. However, the frame rate was only mentioned as 250 Hz at maximum, without mentioning the dataset size at this frame rate [36]. Although utilizing high-end computational hardware can reduce the processing time, an easy-to-deploy, robust, ultrafast projection pipeline can benefit clinical applications such as real-time diagnosis, image-guided surgeries, or treatments. Additionally, it can also serve as a guide for high-quality data acquisition.

This study proposes a novel, robust ultrafast projection pipeline (RUPP) for FD-OCT’s structural and angiography imaging. The RUPP does not require the FT process and only requires OCTA reconstruction on 2D arrays, which can reduce the processing time significantly. Therefore, the proposed pipeline can be used in many potential applications, such as real-time diagnosis, image-guided surgeries, and image-guided treatments. 

## 2. Materials and Methods

### 2.1. Mathematics Explanations

**Figure 1:**
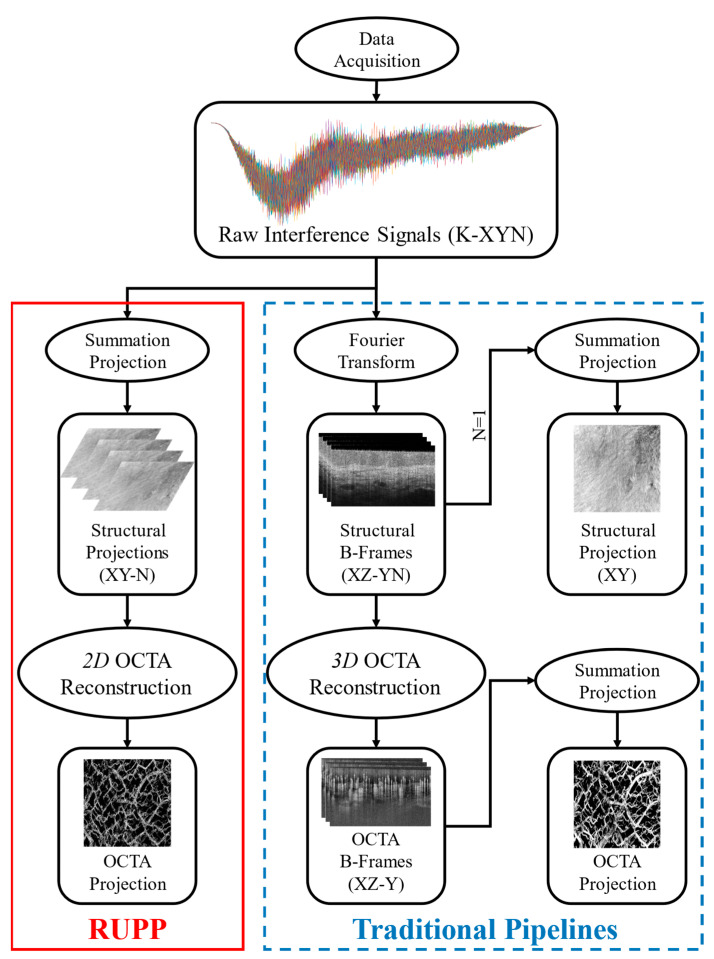
The processing flow diagram of the proposed RUPP (red solid box) and the traditional projection methods (blue dashed box) for FD-OCT structural and angiography imaging.

The flow diagram for the proposed RUPP and the traditional projection methods is shown in Figure 1. A number of raw interference signals would be acquired by the FD-OCT systems. These signals are all of the A-lines in one acquisition, which can be allocated into K-X-Y-N dimensions, where K is the axis of wavenumbers, X and Y are the two lateral axes, and N is the repeated number in the temporal axis. The traditional pipelines to generate projections are shown in Figure 1 as well, in which the FT, 3D OCTA reconstruction and summation projection would be required.

On the other hand, the RUPP directly uses the electromagnetic interference signal from OCT acquisition without Fourier transform to generate structural projections first. For each A-line, the electromagnetic interference signal can be represented as $E\left(k\right)$, where k is the index of the interference wavenumbers. And the intensity signal of each A-line I(z) can be calculated by:

(1) $$I\left(z\right)=F\{E\left(k\right)\},$$

where z is the index of the intensity signal and F is the symbol for Fourier transform. According to the Poisson summation formula, the summations of a signal before and after the Fourier transform are equal [37]. Although the Poisson summation formula can only be applied to the discrete digital signals that are periodic [37,38], the electromagnetic interference signals $E\left(k\right)$ consist of multiple periodic sine/cosine signals with different frequency components, which would be transferred into different depths in the intensity signals I(z). Therefore, the interference signals in FD-OCT can be considered periodic signals, and, as a result, the summations of the interference and intensity signals are equal. Since the summation of the intensity signals can be used to generate the structural projections, the summations of the interference signals, $E\left(k\right)$, can also generate structural projections, Proj, which is shown as:

(2) $$Proj_{x,y}=\sum_{z=1}^{Z}I_{x,y}\left(z\right)=\sum_{k=1}^{K}E_{x,y}\left(k\right),$$

where x and y are the coordinates of each A-line and Z and K are the total numbers of intensity depth index and interference wavenumbers in one A-line, respectively. Therefore, a simple summation of the interference signals can generate structural projections. 

For OCTA projection, the same processes of structural projection generation are needed. By repeatedly scanning the same location, a stack of the structural projections can be provided as:

(3) $${\left[Proj\right]}_{N}=\left[Proj_{1},Proj_{2},\ldots ,Proj_{N}\right],$$

where N is the number of repeated scans. An ensemble of samples from the same scanning location can be modeled as the sum of three components, a clutter component $Proj_{c}$ mainly consisting of static tissue signal, an angiography component $Proj_{a}$, and noise $Proj_{noise}$, which can be shown as:

(4) $${\left[Proj\right]}_{N}=Proj_{c}+Proj_{a}+Proj_{noise}.$$

To extract $Proj_{a}$, several OCTA reconstruction algorithms can be used. The proposed pipeline utilized two algorithms, speckle variance (SV) [33] and ED [39]. As ED was reported as a superior algorithm to achieve theoretical maximum clutter suppression [6], ED was used firstly to extract the angiography component from the repeated projections. Because the clutter component is the dominant component, the signal correlation matrix $\hat{C_{Proj}}$ can be estimated by:

(5) $$\hat{C_{Proj}}=1/N\cdot Proj\cdot Proj^{H},$$

where H is the Hermitian transpose operation. The eigenvectors and eigenvalues can be estimated through decomposition:

(6) $$\hat{C_{Proj}}=M\Lambda M^{H}=\sum_{i=1}^{N}\lambda \left(i\right)e\left(i\right)e{\left(i\right)}^{H},$$

where M is the unitary matrix of eigenvectors $\left[e\left(1\right),e\left(2\right),\ldots ,e\left(N\right)\right]$ and Λ is the diagonal matrix of eigenvalues $\left[\lambda \left(1\right),\lambda \left(2\right),\ldots ,\lambda \left(N\right)\right]$ that are sorted decreasingly. Then, the angiography component can be extracted by:

(7) $$Proj_{a}=\left[I_{d}-\sum_{i=1}^{r}e\left(i\right)e{\left(i\right)}^{H}\right]\cdot {\left[Proj\right]}_{N},$$

where $I_{d}$ is the identity matrix and r is the number of removed eigenvectors. The number of eigenvectors that are removed can be set manually or automatically. In this study, r was set automatically to be the number of eigenvalues that were larger than the average of all eigenvalues, as those removed eigenvalues and corresponding eigenvectors represented the dominant component, which was the clutter component contributed by static tissue.

On the other side, as an efficient intensity-based OCTA reconstruction algorithm, SV was also utilized to extract the angiography component, $Proj_{a}$. SV [33] has a simplified process, which compares frames to the average image, as shown below:

(8) $$Proj_{a}=1/N\cdot \sum_{n=1}^{N}{\left(Proj_{n}-Proj_{mean}\right)}^{2},$$

where $Proj_{mean}$ is the average of all stacked structural projections ${\left[Proj\right]}_{N}$ and $Proj_{n}$ is the nth structural projection in the stack.

**Figure 2:**
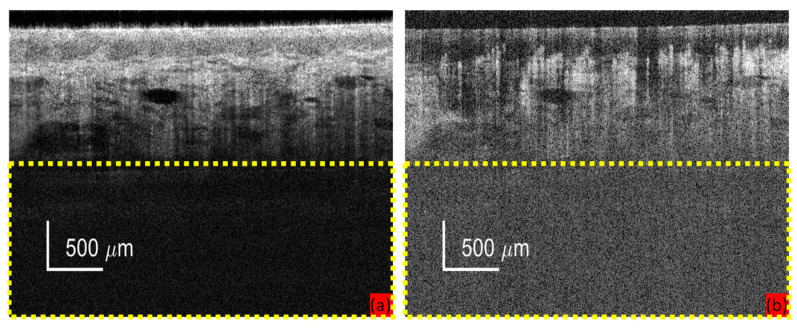
An example of cross-sectional OCT (**a**) and OCTA (**b**) images. The deeper half of the imaging depth was highlighted with yellow dashed boxes.

To further improve efficiency, an optional down-sampling procedure can be added at the data acquisition stage when the targeted information of the acquisition is within the superficial depths. Specifically, the imaging depth of an OCT system is limited by the penetration depth of light, not the theoretical imaging depth of the system setup [40]. Shown in Figure 2 are examples of cross-sectional OCT (a) and OCTA (b) images. The deeper half of the imaging depth was highlighted with yellow dashed boxes. The useful information was often attenuated significantly at the deeper half of imaging depth due to the penetration depth of light in dermatological and oral tissue, regardless of the high imaging depth in air provided by the light source manufacturer.

Therefore, the deep region of the intensity signals consists of low-intensity signals or even just background noise, while the deep-intensity signals come from the high-frequency components of the interference signal [4,40]. As a result, the high-frequency components in the electromagnetic interference signal can be ignored during the projection generation step. For the Fourier transform in FD-OCT, the Nyquist–Shannon sampling theorem limits the highest frequency of the electromagnetic interference signal that can be converted [41,42]. Therefore, the down-sampling procedure on OCT interference signals can decrease the Nyquist frequency, which would act as a cut-off threshold for the OCT intensity signals. As a result, the theoretical imaging depth of OCT is reduced. In addition, down-sampling the interference signals would not require additional processing costs, as it can be applied during the data acquisition stage. When acquiring the interference signal, the data points can be saved into RAM, VAM, or other storage devices selectively, e.g., only odd-numbered data points being saved, which was applied in this study. As a result, the saved acquisition data would be already down-sampled, which can increase the computational efficiency in Equation (2).

To sum up, the proposed RUPP utilized the Poisson summation formula to skip the Fourier transform and to directly generate the structural projection images. Then, the repeated scans with OCTA reconstruction can provide the angiography projection results. 

### 2.2. Experiment Setup and Participants

The suggested RUPP was assessed by applying the mentioned processing techniques to the data gathered from a swept-source OCT system. The light source of this system operated at a sweeping rate of 400 kHz, featuring a central wavelength of 1300 nm and a bandwidth of 100 nm. In total, 22 raw datasets were collected from the hand skin (9 datasets, 6 participants) and oral lip skin (13 datasets, 2 participants) of 8 healthy participants, which was reviewed and approved by the Research Ethics Committee of the University of Dundee (UOD-SSREC-RPG-BioEng-2022-001 and UOD-SSREC-RPG-BioEng-2022-003). All participants had to give their informed consent before entering the lab for the data collection and the informed consent of the participants was obtained for the data collected in this article. The collected data was anonymized and the participants’ identification was removed. The dimensions of each raw interference dataset for assessed techniques were 768 × 400 × 400 × 4 (K × X × Y × N in pixels). The RUPP and other traditional methods (aED [20], bED [21], wED [30], and traditional SV [33]) were applied to the datasets. Particularly, the RUPP was optimized by comparing between the RUPP using ED (RUPP-ED) and SV (RUPP-SV), additionally with or without the optional down-sampling. The software used for processing was MATLAB R2022b (The MathWorks, Inc., Natick, MA, USA), and the computational hardware consisted of a 12th Gen Intel^®^ Core™ i9-12900K CPU (Intel Corporation, Santa Clara, CA, USA), an NVIDIA GeForce RTX 3060 GPU (NVIDIA Corporation, Santa Clara, CA, USA), 32 GB of DDR5 RAM (Samsung Electronics Co., Ltd., Suwon, Gyeonggi Province, Republic of Korea), and a Western Digital PC SN810 Solid-State Drive (Western Digital Corporation, San Jose, CA, USA).

### 2.3. Evaluation Methods

For the above comparisons, the peak signal-to-noise ratio (PSNR), structural similarity (SSIM) index, and processing time were used as the metrics. In the PSNR and SSIM computation, the input image is mathematically compared with the ground truth. However, for in-vivo studies, the ground-truth image is often hard to acquire. Therefore, the *en-face* projections generated from the high-repeated acquisitions were considered as the ground truth. Specifically, for structural projection generation, each A-line signal in the ground truth, $I_{GT}$ was calculated from the average value of multiple repeats, as in

(9) $$I_{GT}\left(z\right)=average\left({\left[I\left(z\right)\right]}^{N}\right),$$

where ${\left[I\right]}^{N}$ is a set of the N-repeated A-line signals, and average() is to calculate the average value of the input. In this study, N is 12 for the ground truth datasets. Using the average values from repeated acquisition at the same location was shown to have a better performance on noise reduction of structural OCT projections [43]. After calculating all ground-truth A-lines, the structural projection ground truth can be generated using the same method, which was shown in Equation (2). On the other side, the ground-truth OCTA projection was generated from 12-repeated traditional wED pipeline [30] due to two reasons: (a) the traditional wED pipeline was proved to have outstanding imaging quality, especially when countering motion artifacts [30]; (b) 12 repeated acquisitions at the same locations with the ED algorithm can provide results with high performance on angiography extraction [44,45].

With the ground-truth OCT and OCTA projections generated, PSNR values can be derived from:

(10) $$PSNR=10log_{10}\left(Proj_{maximum}^{2}/MSE\right),$$

where $Proj_{maximum}$ represents the input image’s maximum value and MSE indicates the mean square error (MSE) between the input and ground-truth images [46,47]. The MSE [48] is determined by:

(11) $$MSE=\left(\sum_{x=1}^{X}\sum_{y=1}^{Y}{\left(Proj_{x,y}-Proj_{GT_{x,y}}\right)}^{2}\right)/\left(X\cdot Y\right),$$

where X and Y are the total numbers of data points on X-axis and Y-axis of projections, respectively, and $Proj_{GT}$ is the ground-truth projection. Then, the SSIM index can be calculated by:

(12) $$SSIM\left(Proj,Proj_{GT}\right)=C_{l}{\left(Proj,Proj_{GT}\right)}^{\alpha }C_{c}{\left(Proj,Proj_{GT}\right)}^{\beta }C_{s}{\left(Proj,Proj_{GT}\right)}^{\gamma },$$

where $C_{l}$, $C_{c}$ and $C_{s}$, respectively, are the comparison measurements of luminance, contrast, and structure between the two input images, and α>0, β>0, γ>0, which are three weighted parameters of $C_{l}$, $C_{c}$, and $C_{s}$, respectively [49].

## 3. Results

### 3.1. Structural Projections

**Figure 3:**
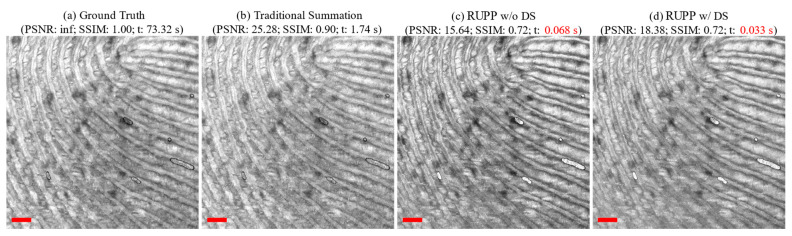
An example (fingertip) of structural OCT projections: (**a**) the ground truth generated from 12-repeat dataset; (**b**) traditional summation projection; (**c**) RUPP without down-sampling; (**d**) RUPP with down-sampling. (t: processing time in seconds; the processing time of RUPP variations are highlighted in red fonts; all red scale bars indicate 500 μm).

The structural OCT projections among the traditional summation projection, RUPP without and with down-sampling were compared with the generated ground-truth structural OCT projections. Figure 3 shows an exemplary dataset, which includes the results generated by all three structural projection techniques.

**Figure 4:**
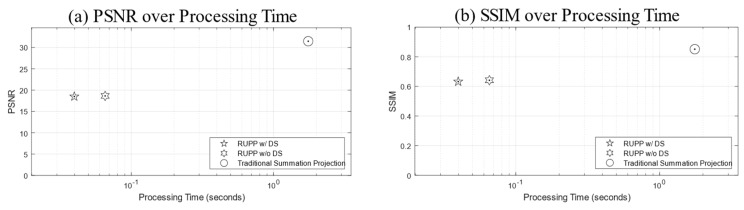
Two plot figures of evaluation metrics over processing time: (**a**) PSNR and (**b**) SSIM over processing time. Note that the horizontal axes (processing time) are all in log scale.

To further evaluate the RUPP methods, the average PSNR and SSIM values were calculated from a total of 22 datasets. Figure 4 shows the relationship between PSNR (a), SSIM (b), and the processing time of the three aforementioned processing techniques.

Additionally, for accurate demonstration with standard deviations, Table 1 shows the PSNR and SSIM values compared to the processing time.

### 3.2. Angiography Projections

**Figure 5:**
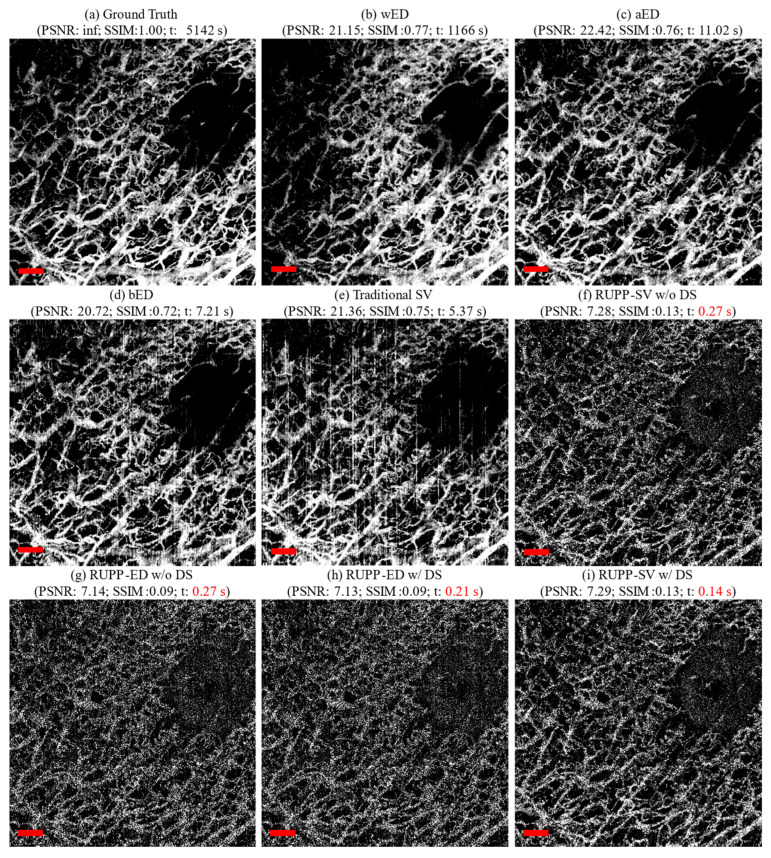
An example of OCTA projections imaging an oral ulcer lesion using various techniques: (**a**) the ground truth generated from 12-repeat dataset, (**b**) wED, (**c**) aED, (**d**) bED, (**e**) traditional SV, (**f**) RUPP-SV without down-sampling, (**g**) RUPP-ED without down-sampling, (**h**) RUPP-ED with down-sampling, and (**i**) RUPP-SV with down-sampling. (t: processing time in seconds; the processing time of RUPP variations are highlighted in red fonts; all red scale bars indicate 500 μm).

For OCTA projections, an extensive evaluation of RUPP angiography projection methods was conducted using a comprehensive approach that involved the application of the aforementioned various methods, namely wED, aED, bED, SV, RUPP-ED with and without down-sampling, and RUPP-SV with and without down-sampling to generate OCTA projections for comparison. The outcomes of these OCTA projection techniques were compared against the high-repeated ground truth, with three evaluation metrics, PSNR, SSIM, and processing time. Figure 5 shows an exemplary dataset of an oral ulcer lesion that was used by various OCTA projection generation techniques.

**Figure 6:**
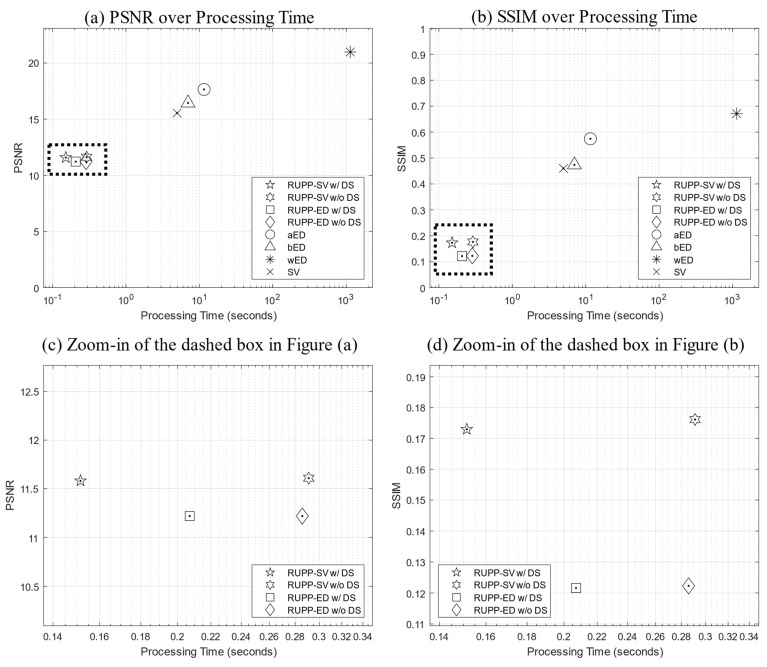
Plot figures of evaluation metrics, (**a**) PSNR and (**b**) SSIM over processing time. (**c**,**d**) are the zoom-in plots of the dashed boxes in (**a**,**b**), respectively. Note that the horizontal axes (processing time) are all in log scale.

In total, 22 datasets were used to produce the average PSNR, SSIM, and processing time values for a robust and objective assessment, which is shown in Figure 6.

Additionally, for accurate demonstration with standard deviations, Table 2 shows the PSNR and SSIM values compared to the processing time.

## 4. Discussion

This study proposed a novel pipeline, RUPP, with variations for both OCT and OCTA projection generation applications. In addition, RUPP is easy to deploy and robust and can be applied to all FD-OCT systems. The RUPP can avoid the Fourier transform process, which would be required in FD-OCT systems. Therefore, the RUPP can significantly reduce the computational cost, which can enable the projection generation as a real-time process. With real-time OCT and OCTA projections, it would be possible to realize real-time diagnosis and surgeries and to improve data acquisition quality by accurate localization and avoiding artifacts. Although SD-OCT systems were not used in this study, theoretically RUPP would also apply to SD-OCT systems because RUPP directly processes the raw interference signals of FD-OCT.

Firstly, RUPP can generate structural OCT projections with extremely low computational costs. With 22 datasets on average, RUPP only took 0.076 s while the traditional summation projection method needed 1.76 s. RUPP reduced the processing time on the same datasets and hardware by 27 times. With the optional down-sampling technique, RUPP can almost halve the processing time, reducing it to 0.040 s, which is 44 times faster compared to the traditional method. For OCTA projections, this study compared eight methods including both traditional techniques and RUPP variations. The SV and ED algorithms were applied to the RUPP to generate OCTA projections. RUPP-SV with down-sampling achieved the fastest processing (reduced the processing time by 7518, 77, 46, and 33 times compared to the traditional wED, aED, bED, and SV techniques, respectively) while RUPP-SV without down-sampling preserved a higher imaging quality than RUPP-ED in terms of PSNR and SSIM values. With a realistic and practical data acquisition size, 768 × 400 × 400 × 4 (K × X × Y × N in pixels) without resorting to an artificially small data size to exaggerate performance benefits, RUPP-SV with down-sampling only required 0.15 s. With this low processing time, the real-time OCTA projection can be achieved or improved significantly. In addition, higher-end computing equipment and parallel processing can potentially further decrease the processing time. However, when not applying the optional down-sampling process, RUPP-ED would require less processing time than RUPP-SV, while RUPP-SV can achieve better imaging quality. Although ED was reported as a superior OCTA reconstruction algorithm in several studies [20,21,30], SV had better performance in terms of PSNR and SSIM values for the RUPP. The reason for ED’s low performance in imaging quality could be that ED is an OCTA reconstruction algorithm based on both the magnitude and phase of the input signals, yet there would be no phase information for the RUPP as the FT process is avoided.

The tradeoff between processing time and imaging quality can also be found for RUPP. The PSNR and SSIM values of RUPP had some decreases in general compared to the traditional projection methods. For structural projections, although the PSNR and SSIM values were, respectively, 41% and 25% lower than the traditional method, the projection results are visually acceptable for feature assessments, especially for the fingerprint on the fingertip in Figure 3. For the angiography projections, the most efficient variant, RUPP-SV with down-sampling, had a 45% and 74% lower PSNR and SSIM values compared to the best-performing traditional projection method, wED. However, the proposed RUPP, as an ultrafast algorithm, is designed for quick identification and localization of the region of interest or real-time diagnosis and treatment monitoring. From visual observation in Figure 5, the vessel structure and the boundary between the normal tissue and the oral ulcer lesion were still well preserved, meaning that RUPP still fits with the above purposes. The reason for low PSNR and SSIM values could be that the blood vessels in RUPP results were not fully solid (i.e., there were some small black “holes” inside vessels in Figure 5), which may preserve good vessel structures for visual observation but would not be friendly for pixel-wise evaluation methods like PSNR and SSIM. In addition, some processing methods like thresholding, erosion, and dilation [50] can potentially improve the RUPP’s imaging quality. Besides, an artificial neural network could be a method to enhance the quality of the vasculature image from the RUPP while maintaining a short processing time [44,51]. With RUPP’s compression on the raw interference signal, the depth information of the vasculature network would be lost. Although the traditional projections do not contain depth information either, the depth information can be extracted during the processing. However, RUPP results can serve as a guide and boundary detection during data acquisition. For example, alignment, registration, or operator-guided survey scans can benefit from RUPP’s fast processing. With only 0.040 and 0.15 s for structural and angiography imaging, RUPP has great potential for real-time projection imaging for FD-OCT. On the other hand, it is possible that the RUPP can be limited by the FD-OCT system’s hardware setup. The RUPP is purely on the software side, while OCT systems would require efficient integration between the software and hardware. For example, the optional down-sampling process may not be possible to deploy depending on the data acquisition device. However, this study provides a few variations of the RUPP, which can be deployed easily. This study can also serve as a guide for utilizing the RUPP in the most efficient way.

## 5. Conclusions

We proposed a novel RUPP for both structural and angiography projection generation, including a few variations for OCTA projections. This RUPP can be easily deployed to all FD-OCT systems. The key advantage of the RUPP is the extremely low processing time. For the structural projections, the RUPP required 27 times less processing time than the traditional projection method. For OCTA projections, RUPP-SV with down-sampling was the most efficient variation of the RUPP, which required up to 7518 times less processing time compared to traditional methods. Although the imaging quality was also decreased, the low processing time makes the RUPP have the significant potential to contribute to real-time structural and angiography projections, which in turn can be useful for real-time surgeries or treatments, quick prognosis, and data acquisition guidance.

## Figures and Tables

**Table 1:** Comparison among various structural OCT projection techniques.

Projection Generation Methods	Evaluation Metrics		
	PSNR	SSIM	Processing Time (s)
Traditional summation projection	31.45 ± 4.45	0.85 ± 0.08	1.76 ± 0.02
RUPP w/o DS ^a^	18.63 ± 4.03	0.64 ± 0.09	0.066 ± 0.0025
RUPP w/DS ^b^	18.49 ± 4.11	0.63 ± 0.09	0.040 ± 0.0054

^a^ RUPP without down-sampling; ^b^ RUPP with down-sampling.

**Table 2:** Comparison among various OCTA projection techniques.

Projection Generation Methods	Evaluation Metrics		
	PSNR	SSIM	Processing Time (s)
wED	20.98 ± 1.34	0.67 ± 0.059	1139 ± 5.10
aED	17.64 ± 2.25	0.57 ± 0.076	11.59 ± 0.23
bED	16.44 ± 1.29	0.47 ± 0.085	7.01 ± 0.22
SV	15.55 ± 1.72	0.46 ± 0.095	4.97 ± 0.19
RUPP-SV w/o DS ^a^	11.61 ± 1.52	0.18 ± 0.036	0.29 ± 0.0060
RUPP-ED w/o DS ^b^	11.23 ± 1.53	0.12 ± 0.021	0.29 ± 0.0098
RUPP-ED w DS ^c^	11.23 ± 1.51	0.12 ± 0.020	0.21 ± 0.0098
RUPP-SV w/DS ^d^	11.58 ± 1.51	0.17 ± 0.036	0.15 ± 0.011

^a^ RUPP-SV without down-sampling; ^b^ RUPP-ED without down-sampling; ^c^ RUPP-ED with down-sampling; ^d^ RUPP-SV with down-sampling.

## Data Availability

The data that support the findings of this article are not publicly available due to ethical restrictions. They can be requested from the correspondence author at c.li@dundee.ac.uk.
